# Cardiac transcriptome profiling of diabetic Akita mice using microarray and next generation sequencing

**DOI:** 10.1371/journal.pone.0182828

**Published:** 2017-08-24

**Authors:** Varun Kesherwani, Hamid R. Shahshahan, Paras K. Mishra

**Affiliations:** 1 Department of Cellular and Integrative Physiology, University of Nebraska Medical Center, Omaha, NE, United States of America; 2 Department of Anesthesiology, University of Nebraska Medical Center, Omaha, NE, United States of America; Virginia Commonwealth University Medical Center, UNITED STATES

## Abstract

Although diabetes mellitus (DM) causes cardiomyopathy and exacerbates heart failure, the underlying molecular mechanisms for diabetic cardiomyopathy/heart failure are poorly understood. Insulin2 mutant (Ins2^+/-^) Akita is a mouse model of T1DM, which manifests cardiac dysfunction. However, molecular changes at cardiac transcriptome level that lead to cardiomyopathy remain unclear. To understand the molecular changes in the heart of diabetic Akita mice, we profiled cardiac transcriptome of Ins2^+/-^ Akita and Ins2^+/+^ control mice using next generation sequencing (NGS) and microarray, and determined the implications of differentially expressed genes on various heart failure signaling pathways using Ingenuity pathway (IPA) analysis. First, we validated hyperglycemia, increased cardiac fibrosis, and cardiac dysfunction in twelve-week male diabetic Akita. Then, we analyzed the transcriptome levels in the heart. NGS analyses on Akita heart revealed 137 differentially expressed transcripts, where Bone Morphogenic Protein-10 (*BMP10*) was the most upregulated and hairy and enhancer of split-related (*HELT*) was the most downregulated gene. Moreover, twelve long non-coding RNAs (lncRNAs) were upregulated. The microarray analyses on Akita heart showed 351 differentially expressed transcripts, where vomeronasal-1 receptor-180 (*Vmn1r180*) was the most upregulated and WD Repeat Domain 83 Opposite Strand (*WDR83OS*) was the most downregulated gene. Further, miR-101c and H19 lncRNA were upregulated but Neat1 lncRNA was downregulated in Akita heart. Eleven common genes were upregulated in Akita heart in both NGS and microarray analyses. IPA analyses revealed the role of these differentially expressed genes in key signaling pathways involved in diabetic cardiomyopathy. Our results provide a platform to initiate focused future studies by targeting these genes and/or non-coding RNAs, which are differentially expressed in Akita hearts and are involved in diabetic cardiomyopathy.

## Introduction

Insulin2 heterozygous (Ins2^+/-^) Akita mice is a spontaneous genetic model for type1 diabetes mellitus (T1DM) [1, 2]. They develop diabetic phenotype at the age of three-four weeks, and male mice show more severe phenotype than female mice [1]. At ten-week, male mice have elevated levels of blood glucose and glycohemoglobin (HbA1c) and reduced levels of insulin [3], the hallmarks of T1DM phenotype. They manifest progress in the diabetic complications including cardiomyopathy with aging, and die within 305 days, which is less than the half of the life span of non-diabetic mice (nearly 690 days) [1, 4]. At the age of twelve weeks, their blood glucose levels are dramatically high and they develop diabetic cardiomyopathy [5, 6]. Previously, we reported the differential expressions of miRNAs in twelve-week male Akita heart [7]. However, the roles of cardiac transcriptomes (genes and non-coding RNAs) of Akita mice on cardiomyopathy/heart failure was unclear. In the present study, our goal was to determine the differential expressions of cardiac transcriptomes of Akita and to assess the potential roles of altered transcriptomes in molecular signaling leading to cardiomyopathy/heart failure.

Microarray is a widely used method to profile differentially expressed known genes. A more advance method to profile global transcriptome is next generation sequencing (NGS). NGS is a RNA-sequencing based technique used for finding out new genes and noncoding RNAs in pathological condition [8]. In the present study, we used both microarray and NGS methods to evaluate upregulated or downregulated transcriptomes in Akita heart. We used Ingenuity Pathway Analyses (IPA), a bioinformatics tool, to determine the potential roles of altered transcriptomes in Akita heart in various signaling pathways involved in diabetic heart failure.

## Materials and methods

### Animals

**Fig 1:**
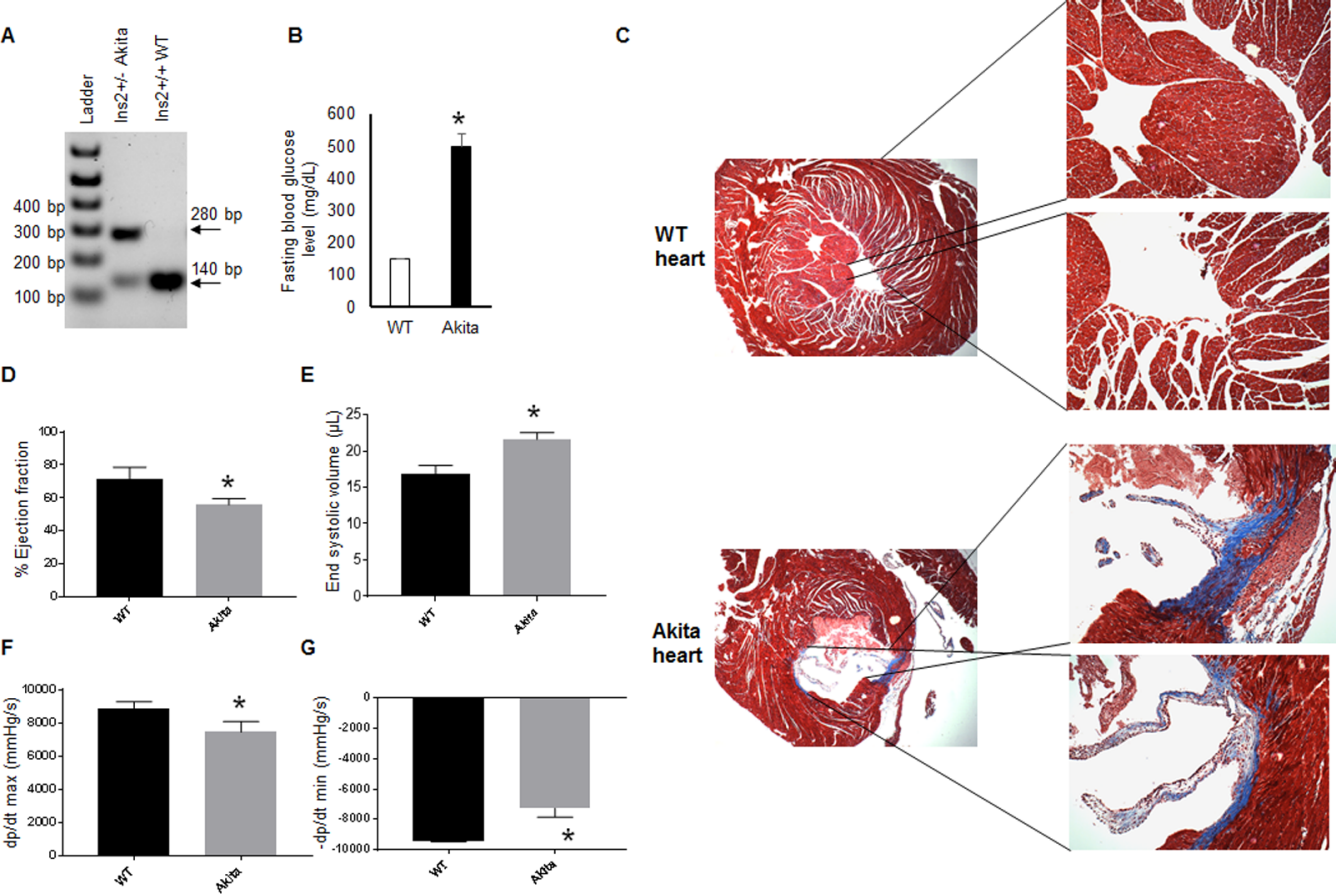
Genotyping and validation of hyperglycemia in Akita mice. (A). Genotyping of WT (Ins2^+/+^) and Akita (Ins2^+/-^) mice. Akita mice have two bands of mutant (280 base pair) and WT (140 base pair) alleles. (B) Fasting blood glucose measurement in WT and Akita mice showed significant upregulation of blood glucose levels in Akita. Values are mean ± SEM. N = 3. (C) Representative Masson Trichrome staining of heart sections of WT and Akita mice showing increased collagen deposition (blue color) in Akita hearts. (D) Hemodynamic analyses from the pressure-volume loop study. The percentage ejection fraction was decreased in Akita. (E) End systolic volume was increased in Akita. (F-G) Left ventricular pressure over time during systole (F) and diastole (G) was decreased in Akita heart. Values are mean ± S. E. N = 3. “*” represents *p*<0.05.

We procured Ins2^+/-^ Akita and C57BL/6J mice from the Jackson Laboratory (Bar Harbor, ME, USA). These mice were bred in animal facility of the University of Nebraska Medical Center. Akita and its normoglycemic sibling (WT) mice were identified by genotyping and blood glucose levels (Fig 1). Twelve-week male Akita and WT mice were used in the present study. Mice were housed in the animal facility of the University of Nebraska Medical Center, and food and water were provided to them ad libitum. This study was approved by the Institutional Animal Care and Use Committee of the University of Nebraska Medical Center.

### Mouse genotyping

We used a small piece of ear tissue and extracted DNA using a kit protocol (Qiagen, USA, catalogue # 69506). We followed our previously published protocol for genotyping [9]. In brief, two primers (forward: 5' TGC TGAT GCC CTG GCC TGCT 3' and reverse: 5' CAC ATA TGC ACA TG 3') were used to amplify the DNA. The PCR protocol was pre-heating at 94°C for 3 min, followed by twelve cycles of 94°C for 20 sec, 64°C for 30 sec with a 0.5°C decrease per cycle and 72°C for 35 sec, then twenty-five cycles of 94°C for 20 sec, 58°C for 30 sec and 72°C for 35 sec, and finally 2 min hold at 72°C and cooling and storage at 4°C. The PCR products were restriction digested and were electrophoresed using 2% agarose gel and bands were imaged using ChemiDoc XRS instrument with Image Lab 4.1 software (Biorad Laboratories, USA). The mutated allele shows a band of 280 base pair, whereas the WT allele shows a band of 140 base pair.

### Hemodynamic measurements

We performed pressure-volume loop measurement using Scisence instrument (ADV 500 system, Transonic, USA). Mice were anaesthetized using 1–2% isoflurane, and then intubated and connected to a ventilator. We used open chest method where probe was inserted directly into the apex. We created fine needle-size hole in the LV using a 27-gauze needle and inserted the probe quickly into it. The probe was adjusted and maintained in a position, where it did not touch the ventricular wall. It was ensured by observing the pressure-volume loop formation. There was no significant change in the heart rate, while collecting the pressure-volume loop data for the different mice groups.

### Masson Trichrome staining

To determine cardiac fibrosis, we performed Masson Trichrome staining using paraffin sections (5 μM thickness) of WT and Akita hearts. Standard kit protocol was followed. We used tissue culture core facility of our university for Masson Trichrome staining.

### RNA isolation, quality assessment and QPCR

Heart tissue was isolated from WT and Akita mice and snap frozen for RNA isolation. We used *mir*Vana miRNA isolation kit (Thermo Fisher Scientific, USA, catalogue # AM1560) for RNA isolation following the protocol of the kit. RNA quality and concentration were measured by NanoDrop 2000c spectrophotometers (Thermo Fisher Scientific, USA). The integrity of RNA was ensured by the Bioanalyzer from the DNA Sequencing Core Facility of the University of Nebraska Medical Center. For qPCR, we used standard protocol as elaborated elsewhere [10]. The primer sequences were: Angptl4, forward: 5´CCTGTGGTAA CGCTTGTC3´, reverse: 5´GAGGTCTATCTGGCTCTGAA 3´; Hmgcs2, forward: 5´AGCAGTGACAAACAGAACAA3´, reverse: 5´GCAGAGTGGTGAGAGAGAAG3´; and PDK4, forward 5´AGATGCCTTTGAGTGTGC3´, and reverse 5´CTTTTCCCAA GACGACAGT3´.

### Microarray

We used 150ng RNA and labelled it with WT Plus labelling kit from Affymetrix. These labelled RNAs were used for complementary RNA (cRNA) synthesis. To proceed for microarray, 10 μg of cRNA was hybridized for 16 hours at 45°C on a GeneChip Mouse Gene 2.0 ST Array. The GeneChips were washed and stained in the Affymetrix Fluidics Station 450, and then scanned using the GeneArray Scanner 3000 7G. Microarray was performed in the DNA Sequencing Core Facility at the University of Nebraska Medical Center. The data were analyzed with Expression Console and TAC software from Affymetrix using default analysis settings. IPA core analyses was performed on differentially expressed transcriptomes with a twofold change cutoff. A *p*-value < 0.05 was used as statistically significant. The sample size was three mice per group.

### Genomic library preparation, quality assessment and sequencing

We used the University of Nebraska Medical Center DNA Core Facility for performing microarray and NGS. In brief, RNA libraries were prepared using TruSeq RNA sample preparation kit from Illumina. For constructing the library, 1μg of RNA was used. The quality control was assessed by Bioanalyzer and quantification was done by Qubit. For cluster generation, 6 Pico molar starting quantity and o-bot clustering was used, and 100 base single read run was performed on Illumina HiSeq 2500. Illumina HCS v2.05 software was used for base calling. Sequenced reads were trimmed for adaptor sequence and masked for low-complexity or low-quality sequence, and then mapped to mm 10 whole genome using Tophat 2 aligner in base space. Reads Per Kilobase of exon per Megabase (RPKM) of library size was calculated using Cufflinks 2, and differential expression was determined by using Cuffdiff 2.1.1 in BaseSpace. IPA core analysis was performed on differentially expressed transcriptomes, which were qualified for a twofold change cutoff and a *p*-value <0.05. The sample size was three mice per group. Raw and analyzed data were uploaded on GEO website (data set # GSE66577).

### Western blotting

To determine the protein levels of Nebulin and GABARAPL1 in WT and Akita hearts, we used standard Western blotting method as described in details in our earlier publications [7, 10]. For protein estimation, we used BCA method. We used primary antibody of Nebulin and GABARAPL1 from Novus (catalogue NB120-11099) and GeneTex (calatogue GTX132664), respectively.

### Statistical analyses

The blood glucose levels and gene expression through qPCR and Western blotting are presented as mean and standard error of mean (mean ± SEM), whereas genes expression through microarray and NGS are expressed as mean and standard deviation (mean ± SD). Only values with changes twofold or more and a *p*-value <0.05 was scored as statistically significant in microarray and NGS gene analyses. To compare the mean of WT and Akita, Student’s t- test was performed and a *p-*value <0.05 was considered statistically significant and represented by “*”.

## Results

### Validation of Akita mice

Previously, others and we have reported that Akita have high blood glucose [1, 2, 9], reduced plasma insulin and increased HbA1c levels [1, 3]. We have documented that at twelve-week, these mice have dramatically elevated blood glucose levels and they manifest cardiac fibrosis and dysfunction [11, 12]. In the present study, we first validated Akita mice by performing genotyping [9, 12], measuring blood glucose levels, and determining cardiac dysfunction [11]. Our results showed that both WT (140 base pair) and mutant (280 base pair) alleles were present in the Insulin2 heterozygous Akita mice (Fig 1A). The fasting blood glucose levels were extremely high in these mice (Fig 1B). These findings validate the Akita mice phenotype. To determine cardiomyopathy and cardiac dysfunction in these mice, we performed Masson Trichrome staining and hemodynamic measurements by pressure-volume loop study, respectively. The collagen deposition (blue color) was increased in Akita hearts (Fig 1C), showing enhanced cardiac fibrosis. The hemodynamic measurements showed decreased ejection fraction in Akita (Fig 1D), demonstrating cardiac dysfunction. The end systolic volume was increased in Akita (Fig 1E) confirming systolic dysfunction in these mice. Moreover, changes in the left ventricular pressure during systole (Fig 1F) and diastole (Fig 1G) was decreased in Akita, showing impaired cardiac contractility in Akita heart. These results confirm the diabetic phenotype and cardiac dysfunction of Akita mice.

### Differentially expressed global transcriptomes in Akita heart

**Fig 2:**
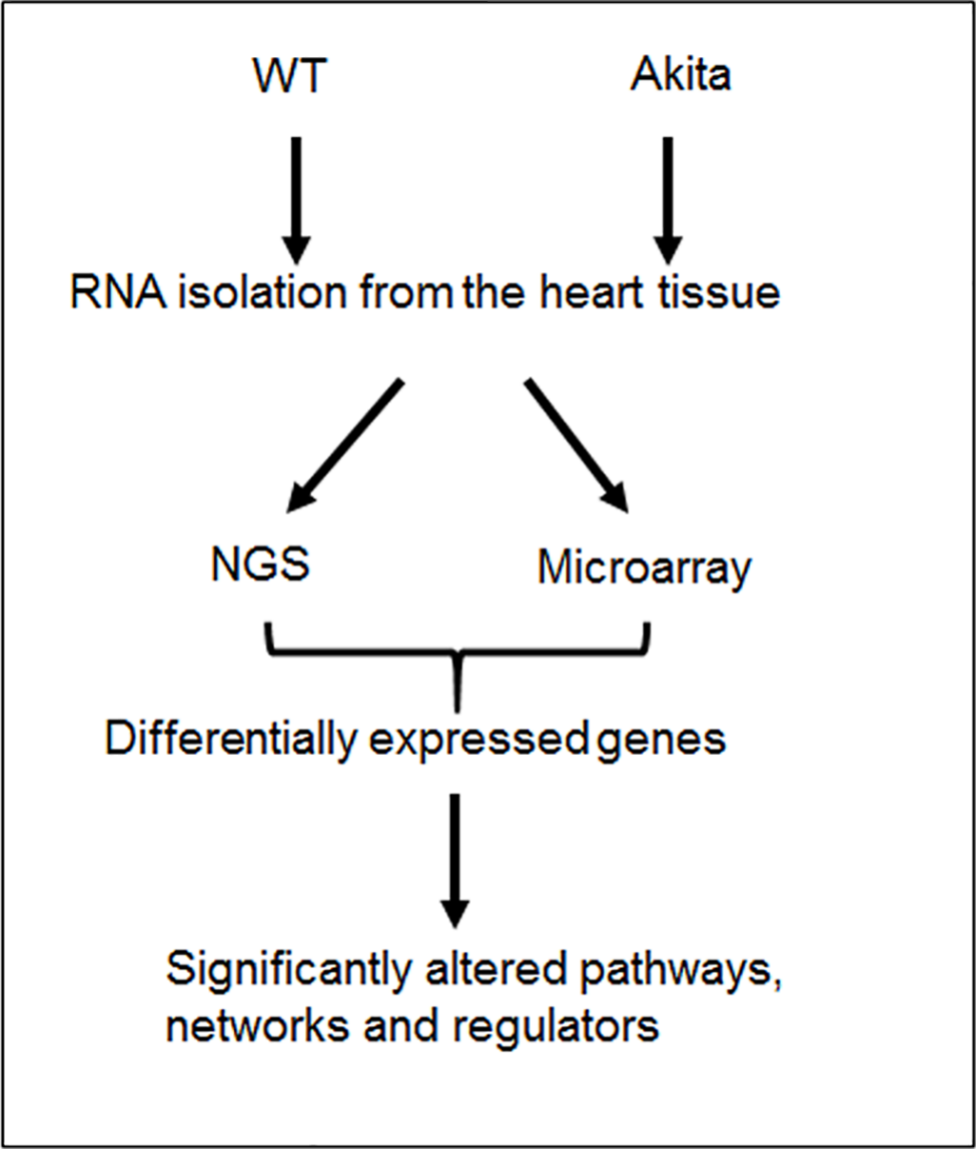
Schematic representation of workflow. Cardiac profiling of Akita mice using next generation sequencing (NGS) and microarray, and evaluating the implications of differentially expressed genes on signaling pathways, using Ingenuity Pathway Analyses (IPA).

**Fig 3:**
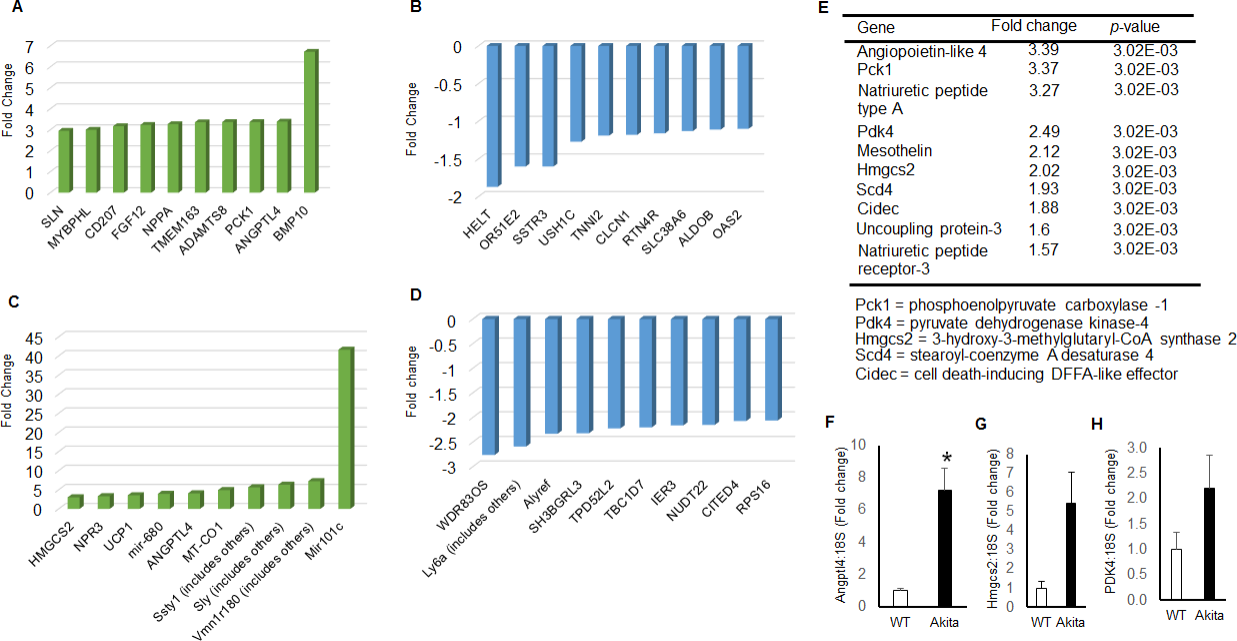
Transcriptome profiling of WT and Akita hearts using next generation sequencing (NGS) and microarray analyses. (A) Top-ten upregulated transcriptome in Akita by NGS analyses. (B) Top-ten downregulated transcriptome in Akita heart by NGS analyses. (C) Top-ten upregulated transcriptome in Akita heart by microarray analyses. (D) Top-ten downregulated transcriptome in Akita heart by microarray analyses. (E) Top-ten upregulated genes in Akita heart common in both NGS and microarray analyses (fold change ≤ 2 or 2 ≥ and *p-*value < 0.05). All the data are represented as fold change of WT hearts. Values are mean ± SD. N = 3. (F-H) Validation of three upregulated genes in Akita heart using an independent experiment and qPCR analyses. Values are represented as mean ± SEM. N = 3.

To determine the differential expression of transcriptome of Akita heart, we isolated total RNA from the whole heart of WT and Akita mice, and performed NGS and microarray assays. The data obtained from NGS and microarray of WT and Akita hearts were analyzed for differentially expressed genes. These genes were used for IPA analyses to determine their implications in heart failure signaling pathways (Fig 2). NGS and microarray analyses from WT and Akita hearts showed 351 and 137 differentially expressed transcriptomes, respectively, in Akita hearts. By using the filter criteria of twofold change (up-, or down-regulation) and *p*< 0.05 value for difference between WT and Akita, we found 135 and 176 differentially expressed transcripts in NGS and microarray analyses, respectively. The raw and analyzed data were uploaded on GEO website (dataset # GSE66577). We tabulated the top-ten upregulated and top-ten downregulated transcriptomes in NGS (Fig 3A and 3B) and microarray (Fig 3C and 3D) analyses using the above filter criteria. The common transcripts that were similarly changed in both NGS and microarray are tabulated in Fig 2E, where Angiopoietin-like-4 (ANGPTL4) was the top upregulated gene. The other upregulated genes in the decreasing order of expression were phosphoenolpyruvate carboxylase -1 (PCK1), natriuretic peptide type A (NPPA), pyruvate dehydrogenase kinase-4 (PDK4), mesothelin (MSLN), 3-hydroxy-3-methylglutaryl-CoA synthase 2 (HMGCS2), stearoyl-coenzyme A desaturase 4 (Scd4), cell death-inducing DFFA-like effector c (CIDEC), uncoupling protein-3 (UCP3), natriuretic peptide receptor-3 (NPR3), and connective tissue growth factor (CTGF) (Fig 3E). We have validated the expression of three upregulated genes (ANGPTL4, HMGCS2 and PDK) by qPCR using different mice groups. The qPCR results of these independent experiments further validate differential expression of genes by NGS and microarray analyses (Fig 3F–3H).

### Differentially expressed non-coding RNAs in Akita heart

**Fig 4:**
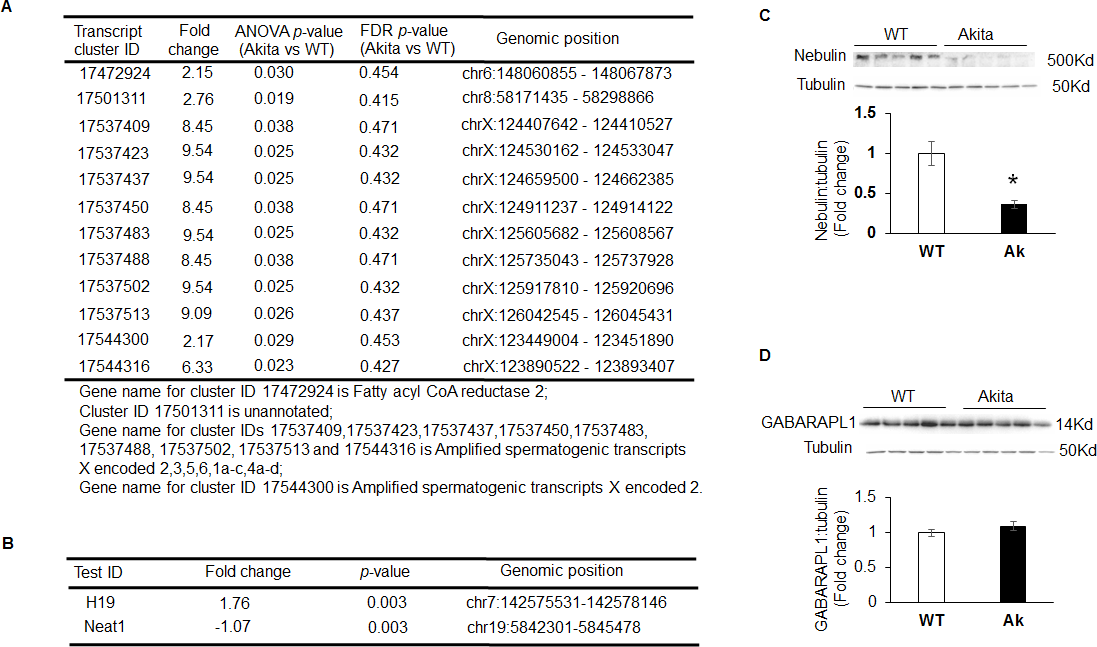
Differentially expressed long-non-coding RNAs (lncRNAs) in Akita heart. (A) NGS analyses showing different lncRNAs upregulated in Akita hearts. (B) Microarray analyses of lncRNAs in Akita hearts. Values are mean ± S. D. N = 3. (C) Representative Western blots and densitometric analyses of bands for Nebulin in WT and Akita hearts. Values are mean ± S. E. N = 5. (D) Representative Western blots and densitometric analyses of bands for GABA (A) receptor associated protein like-1 (GABARAPLK1) in WT and Akita hearts. Values are mean ± S. E. N = 5.

We also evaluated the differentially expressed noncoding RNAs in Akita heart using NGS and microarray methods. By using the filter criteria of twofold change and *p*< 0.05 value, we found a total of sixty-six differentially expressed non-coding RNAs in Akita heart by NGS analyses, where twelve were long non-coding RNAs (lncRNAs) (Fig 4A). We found downregulation of miR-143 in Akita heart by NGS, which was not reported in previous study [7]. In the microarray analyses, we found three differentially expressed non-coding RNAs in Akita heart, where two were lncRNAs. The two differentially expressed lncRNAs were H19 that was upregulated, and Neat1 that was downregulated in Akita heart (Fig 4B). The third non-coding RNA was miR-101c, which was highly upregulated in Akita heart (Fig 3C). MiRNAs mostly inhibit a gene by targeting its 3’UTR of mRNA. One miRNA may have many potential gene targets, whose protein levels may or may not change with up- or down-regulation of the miRNA. We validated miR-101c and miR-143, which were up-, and down-regulated in Akita hearts, respectively, by measuring the protein levels of their target genes. For miR-101c target gene, we measured the protein levels of nebulin, a target gene of miR-101c (www.miRDB.com) that is downregulated in T1DM rat hearts [13]. Our results show decreased levels of nebulin in Akita as compared to WT hearts (Fig 4C) indicating that upregulation of miR-101c may have decreased nebulin in Akita heart. For miR-143 target gene, we measured the protein levels of GABA (A) receptor associated protein like1 (GABARAPL1), whose expression does not change in rat cardiomyocytes after treatment with high glucose [14]. Our results demonstrated that there was no change in the protein levels of GABARAPL1 in Akita hearts (Fig 4D), suggesting that decreased levels of miR-143 do not change the GABARAPL1 levels in Akita hearts.

### IPA analyses of differentially expressed transcriptomes of Akita heart

**Fig 5:**
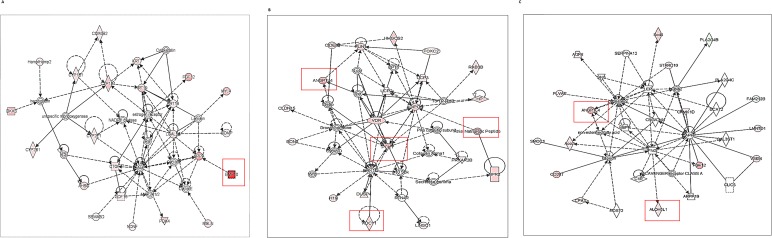
Ingenuity Pathway Analyses (IPA) for key signaling network in Akita heart using differentially expressed genes from NGS analyses. (A-C) The differentially expressed genes obtained from NGS analyses are involved in several key signaling networks in Akita heart.

**Fig 6:**
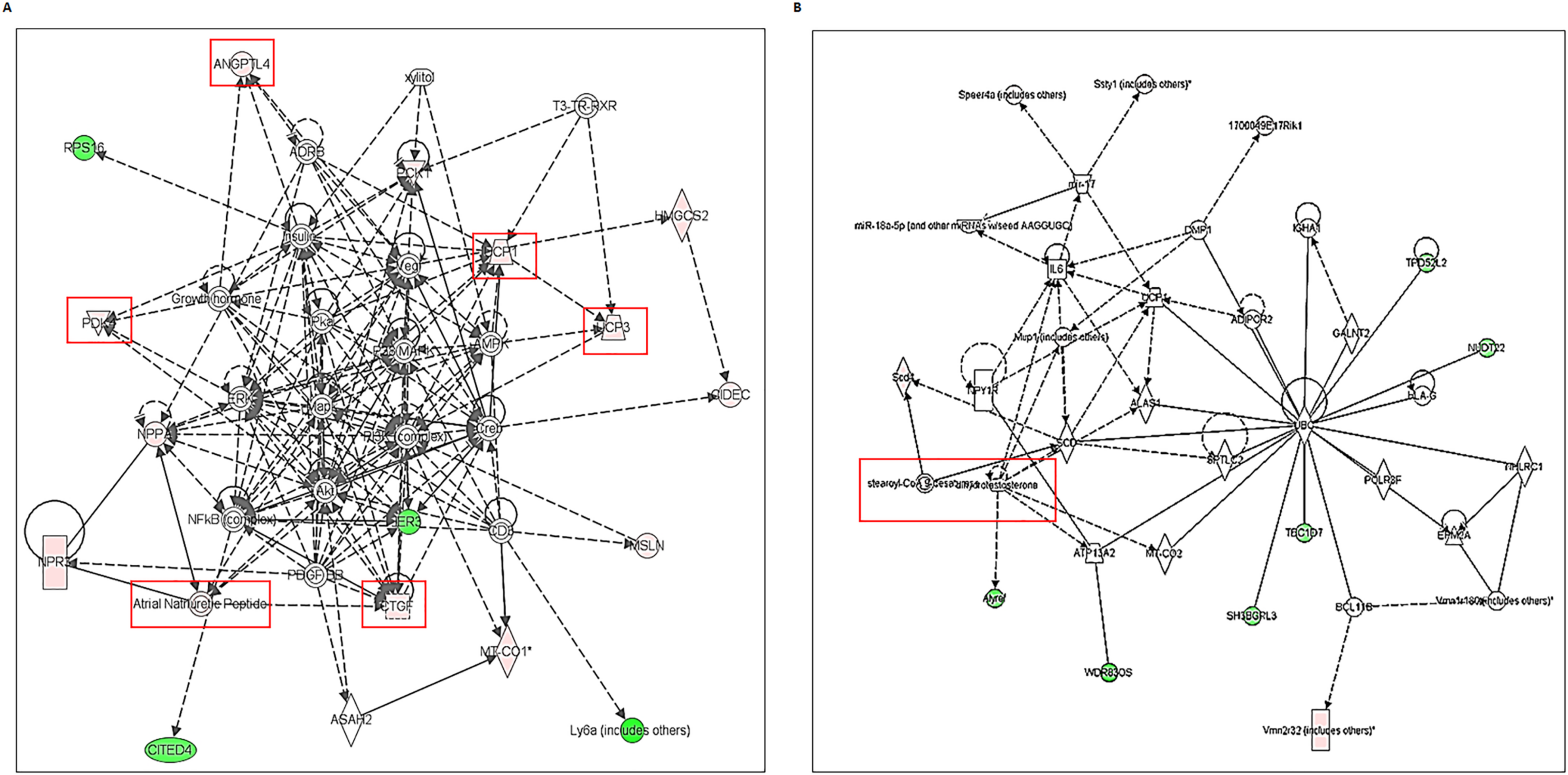
Ingenuity Pathway Analyses (IPA) for key signaling network associated with cardiomyopathy/diabetic heart failure in Akita heart using differentially expressed genes from microarray analyses. (A-B) The differentially expressed genes in Akita heart obtained from microarray analyses are involved in key signaling networks associated with cardiomyopathy/heart failure.

**Fig 7:**
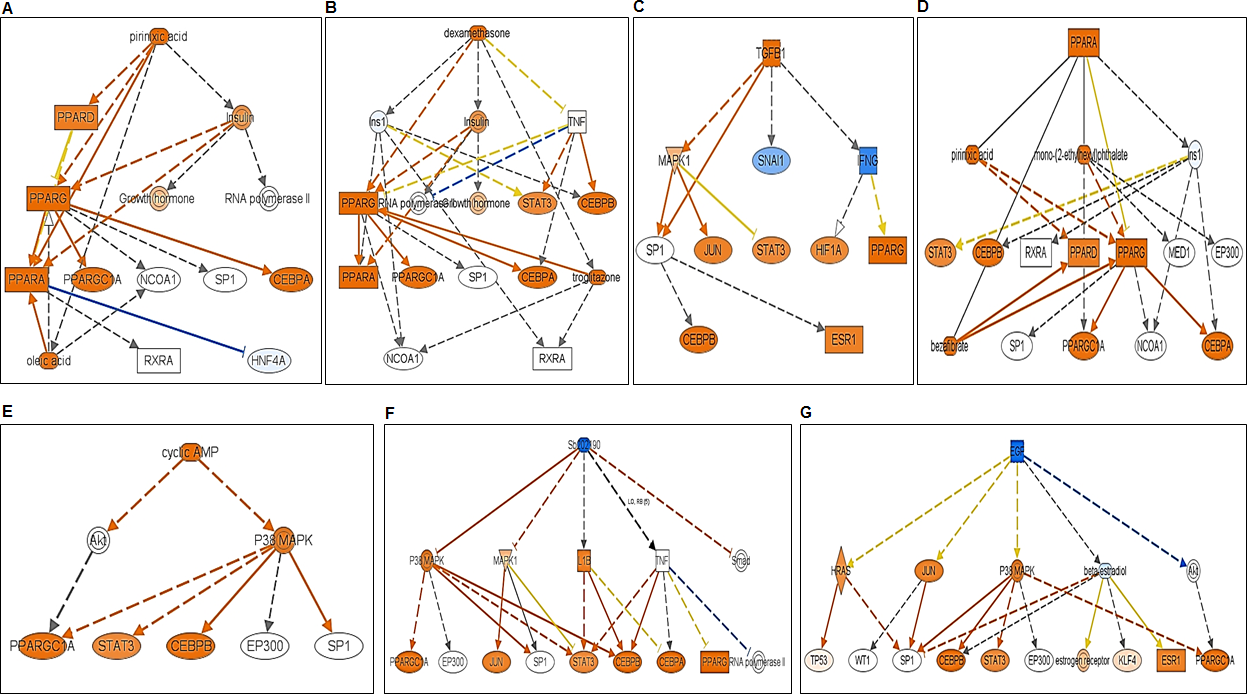
Ingenuity Pathway Analyses (IPA) for key upstream regulators of diabetic heart failure signaling cascade in Akita heart using differentially expressed genes from NGS analyses. (A-E) Upstream regulators activated in Akita heart. (F-G) Upstream regulators suppressed in Akita heart.

**Fig 8:**
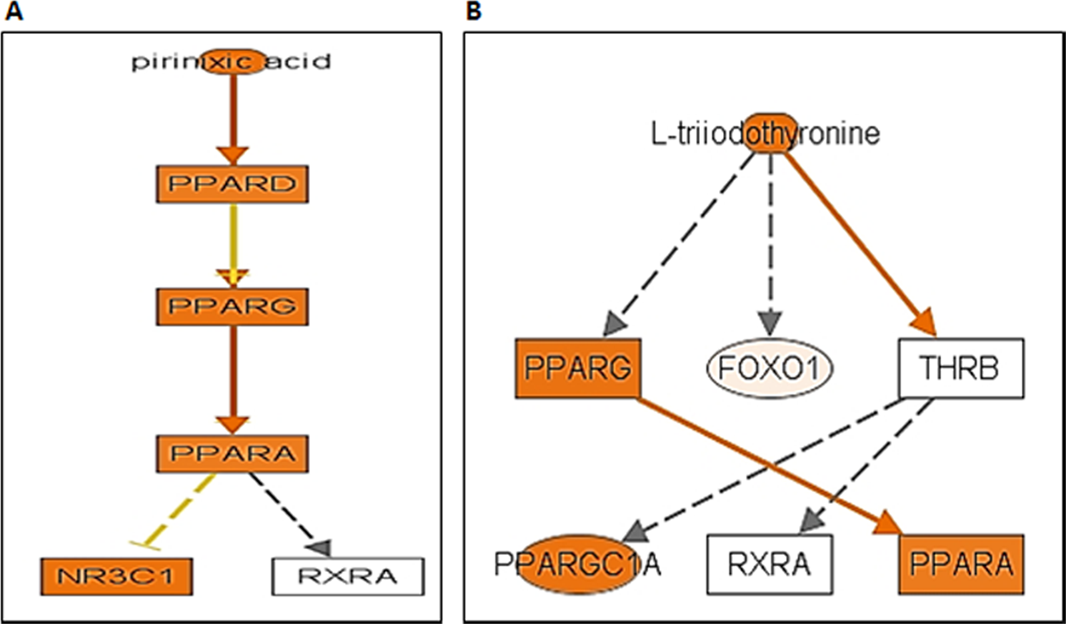
Ingenuity Pathway Analyses (IPA) for key upstream regulators of diabetic heart failure signaling cascade in Akita heart using differentially expressed genes from microarray analyses. (A) Upstream regulators activated in Akita heart. (B) Upstream regulators suppressed in Akita heart.

To determine the association of differentially expressed transcriptomes in Akita heart with a signaling pathway, we performed IPA on NGS and microarray profiled genes. The key pathways influenced by the differentially expressed genes in NGS analyses were calcium signaling, protein kinase A signaling, cyclic AMP-mediated signaling, thyroid receptor activation, and estrogen biosynthesis. The key pathways affected by the microarray profiled differentially expressed genes were cholesterol biosynthesis, ketogenesis, and thyroid receptor activation. The potential signaling network of differentially expressed genes in Akita by NGS and microarray are shown in the Fig 5A–5C, and Fig 6A and 6B, respectively. We found several upstream regulators of signaling pathways in Akita hearts by NGS (Fig 7A–7G) and microarray (Fig 8A and 8B). In NGS analyses, the important upstream regulators activated in Akita hearts are shown in Fig 7A–7E, and important upstream regulators inhibited in Akita are shown in Fig 7F and 7G. Similarly, in microarray analyses the upstream regulators activated in Akita heart are shown in Fig 8A and upstream regulators inhibited in Akita heart are shown in Fig 8B. However, the common upstream regulator in NGS and microarray was peroxisome proliferator-activated receptor alpha (PPARA), which is a nuclear receptor protein that plays a crucial role in ketogenesis.

Overall, our results on global transcriptome profiling of WT and Akita hearts using NGS and microarray revealed many novel candidate genes and non-coding RNAs. We found several common genes that were upregulated in both NGS and microarray analyses, and validated them by qPCR using a separate group of mice. We also used IPA to assess potential roles of differentially expressed genes in Akita heart on various signaling pathways involved in cardiomyopathy/heart failure. In summary, our results revealed many differentially expressed transcriptomes in Akita hearts and their potential roles on various signaling pathways involved in diabetic cardiomyopathy.

## Discussion

Diabetes mellitus (DM) is a complex disease with multiple etiologies and its prevalence is increasing at an alarming rate across the world [15–17]. It is caused either due to insufficient insulin production from pancreatic beta cells (T1DM), or due to insulin resistance (T2DM). DM leads to heart failure [18–20]. However, the underlying molecular mechanisms for diabetic cardiomyopathy/heart failure are largely unknown. Since changes at transcriptome levels initiate the pathological remodeling process that leads to heart failure, it is important to understand differential expressions of transcriptomes of the diabetic heart and determine their implications on heart failure signaling pathways. In the present study, our goal was to determine the global changes at transcriptome levels of Akita heart and to associate these changes with diabetic cardiomyopathy/heart failure. The other objective of this study was to discover new candidate transcriptomes that were not reported in Akita hearts and to discuss their potential roles in diabetic cardiomyopathy. First, we validated the Akita mice model by genotyping (Fig 1A) and measuring blood glucose levels (Fig 1B). Second, we determined pathological remodeling by evaluating cardiac fibrosis (Fig 1C) and cardiac dysfunction by measuring ejection fraction (Fig 1D), end systolic volume (Fig 1E), db/dt mas (Fig 1F) and dp/dt min (Fig 1G). These results support that Akita is a good model to study diabetic cardiomyopathy. Then we evaluated the changes in the transcriptome levels in Akita heart by using microarray and NGS methods (Figs 2–4D). We also analyzed the implications of the differentially expressed transcriptomes in cardiomyopathy signaling cascade by IPA analyses (Figs 5–8B). Our results reveal several novel differentially expressed genes and non-coding RNAs in diabetic Akita heart, and their implications in specific signaling pathways associated with diabetic cardiomyopathy. These findings are crucial for setting a base for future studies that focus on understanding the molecular mechanisms of diabetic heart failure.

Although several model systems have been reported for T1DM in rodents such as non-obese diabetic (NOD) mice—an autoimmune model, chemically-induced diabetes such as streptozotocin-, or alloxan-treated rodents [21], we found Akita as a better model than others because it is a spontaneous, chronic, and genetic model of T1DM. Moreover, it is relevant to humans because mutation in insulin gene causes T1DM in humans [22]. In mouse, there are two insulin genes: Insulin 1 and Insulin 2. Insulin 2 is orthologous to human Insulin. Therefore, mutation of Insulin 2 gene in mouse is relevant to mutation of Insulin gene in humans. These mice show the pathological features of diabetic hearts [1–3, 9]. Therefore, we used Akita mice as a model system for T1DM.

Several changes have been reported at the molecular level in diabetic hearts [15]. Here, we profiled global transcriptome of WT and Akita heart by NGS and microarray methods (Figs 2–4D) to investigate changes at the gene and non-coding RNA levels in Akita heart. NGS is the most advanced RNA-sequencing method for transcriptome profiling and to discover new genes. It is important for identifying tissue specific protein-coding and non-coding transcripts in humans and animal tissues [23–26]. It is also crucial for evaluating differentially expressed genes in heart failure [27–29]. On the other hand, microarray is classically used technique for determining the differential expressions of known transcriptomes. It is valuable for evaluating differential expression of genes in leptin receptor mutant db/db (T2DM) mice [30]. Our results from both microarray and NGS methods reveal many novel transcriptomes that are up-, or downregulated in Akita heart (GEO, data set # GSE66577). The top-ten upregulated genes in Akita heart by NGS analyses are sarcolipin (SLN), myosin binding protein-H like (MYBPHL), langerin (CD207), fibroblast growth factor-12 (FGF12), natriuretic peptide type-A (NPPA), transmembrane protein-163 (TMEM163), ADAM metallopeptidase with thrombospondin type-1 motif-8 (ADAMTS8), phosphoenolpyruvate carboxylase -1 (PCK1), angiopoietin-like-4 (ANGPTL4), and bone morphogenic protein-10 (BMP10) (Fig 3A). The top-ten downregulated genes in Akita hearts by NGS analyses are helt BHLH transcription factor (HELT), olfactory receptor family member -51, subfamily E, member-2 (OR51E2), somatostatin receptor-3 (SSTR3), Usher syndrome-1C (USH1C), troponin-I type-2 (TNNI2), chloride channel voltage sensitive-1 (CLCN1), reticulon-4 receptor (RTN4R), solution carrier family-38, member-6 (SLC38A6), aldolase-B, fructose bisphosphate (ALDOB), 2’-5’-oligoadenylaste synthetase-2 (OAS2) (Fig 3B). NGS analyses also reveal at least twelve lncRNAs that are upregulated in Akita heart (Fig 4A). The differentially expressed transcriptomes obtained from NGS analyses potentially influence several important cardiomyopathy pathways in Akita heart including cAMP-mediated signaling and calcium signaling that play crucial roles in cardiac contractility. The differentially expressed genes in Akita heart have important roles in several signaling pathways associated with diabetic cardiomyopathy (Fig 5A–5C), and several of differentially expressed genes act as upstream regulators for these pathways (Fig 7A–7G).

Microarray analyses on WT and Akita hearts show many differentially expressed transcriptomes in Akita heart (data uploaded on GEO # GSE66577). The top-ten upregulated transcripts in Akita hearts are 3-hydroxy-3-methylglutaryl-CoA synthase 2 (HMGCS2), natriuretic peptide receptor-3 (NPR3), uncoupling protein-1 (UCP1), microRNA-680 (miR-680), angiopoietin-like 4 (ANGPTL4), mitochondrially encoded cytochrome c oxidase-1 (MT-CO1), spermiogenesis specific transcript on the Y-1 (Ssty1), sycp3 like Y-linked (Sly), vomeronasal-1receptor-180 (Vmn1r180) and microRNA-101c (miR-101c) (Fig 3C). The top-ten downregulated transcripts are WD repeat domain 83 opposite strand (WDR83OS), lymphocyte antigen 6 complex, locus A (Ly6a), Aly/REF export factor (Alyref), SH3 binding glutamic acid-rich subunit L3 (SH3BGRL3), tumor protein D52-like2 (TPD52L2), TBC-1 domain family, member 7 (TBC1D7), immediate early response-3 (IER3), nucleotide diphosphate linked moiety X- type motif 22 (NUDT22), Cbp/P300-interacting trans-activator, with Glu/Asp-Rich carboxy-terminal domain, 4(CITED4) and ribosomal protein-16 (RSP16) (Fig 3D). Microarray analyses reveal one upregulated (H19) and one downregulated (Neat1) lncRNA in Akita heart (Fig 4B). IPA analyses on differentially expressed genes in Akita heart by microarray method show several key diabetic cardiomyopathy pathways that are influenced by these genes (Fig 6A and 6B). These genes also act as a potential upstream regulator for important diabetic cardiomyopathy pathways (Fig 8A and 8B).

We found several upregulated genes in Akita hearts that are common in both NGS and microarray analyses. These could be the prime target genes for future molecular analyses on diabetic heart failure in Akita. The top-ten commonly upregulated genes in Akita hearts are ANGPTL4, PCK1, NPPA, PDK4, MSLN, HMGCS2, Scd4, CIDEC, UCP3, NPR3, and CTGF (Fig 3E). We validated four commonly upregulated genes in Akita by an independent qPCR analyses using a separate group of mice (Fig 3F–3H). The common upregulated genes have potentially important roles in various physiological processes. For example, ANGPTL4 is associated with metabolism of lipoproteins [31–35], and is a risk factor for coronary disease [36]. Cardiomyocyte secretory ANGPTL4 protein regulates metabolism during diabetic heart failure [37]. PCK1 plays an important role in glucose metabolism, and gene-nutrient interactions on PCK1 modulates insulin resistance in metabolic syndrome subjects [38]. Mutation of Pck1 gene causes Smith-Magenis Syndrome, where the patients have episodes of hypoglycemia and lactic acidosis [39]. The polymorphism of promoter region of human Pck1 (-232C/G) gene is associated with T2DM [38]. PCK1 is also associated with brain atrophy in multiple sclerosis [40]. NPPA is a hormone involved in regulation of cardiovascular diseases. It has an important role in suppressing murine Th17 cell development via phosphatidylinositol 3´-kinase (PI2K)/Akt signaling [41]. Ablation of Nppa gene causes salt-sensitive hypertension and cardiac hypertrophy in female mice [42]. PDK4 is a mammalian mitochondrial serine kinase protein, which is involved in insulin resistance, a T2DM phenotype [43, 44]. It is also involved in regulation of apoptosis [45]. MSLN is a glycosylphosphatidylinositol-linked glycoprotein, which is highly expressed in mesothelial cells [46]. The overexpression of MSLN is correlated with upregulated matrix metalloproteinase-9 (MMP9) [47], a collagenase associated with cardiac fibrosis [12] and contractile dysfunction [48] in diabetic heart. HMGCS is an enzyme formed by condensation of acetyl-CoA with acetoacetyl-CoA [49]. Deficiency of mitochondrial HMGCS leads to a recessive disorder of ketoacidosis, a disease diagnosed with hypoglycemic hypoketotic coma during fasting period [50]. Murine SCD4 is a rate-limiting enzyme in the biosynthesis of monounsaturated fatty acids. SCD4 is one of the three isoforms of SCD. It is exclusively expressed in the heart and is regulated by leptin and dietary factors [51, 52]. CIDEC (Fst27 in mouse) is a protein involved in lipid metabolism and patients with congenital CIDEC deficiency develop an adverse lipodystrophic phenotype [24]. Mitochondrial UCP3 is a proton carrier. It prevents lipid-induced mitochondrial damage. Reduced levels of UCP3 is found in subjects with T2DM [53]. NPR3 is a receptor for three natriuretic peptides, small peptides involved in regulation of blood volume and pressure, and cardiac function. It clears circulating and extracellular natriuretic peptides through endocytosis, and is inhibited by angiotensin-II, an inducer of hypertension, in vascular smooth muscle cells [54]. Single nucleotide polymorphism in Npr3 is associated with diastolic dysfunction [55], early onset of ischemic stroke [56], and hypertension [57]. CTGF (also known as CCN2) is a matricellular protein [58] that plays important roles in pathobiology of various diseases including fibrotic disease and atherosclerosis [59]. CTGF post-conditioning increases ischemia-reperfusion injury tolerance in murine hearts [60].

Non-coding RNAs are a novel class of regulators for cardiovascular diseases [61, 62]. MicroRNAs (miRNAs) are tiny non-coding RNAs that mostly binds at 3´untranslated region of messenger RNA to modulate gene expression [63, 64]. They are promising therapeutic target for cardiovascular diseases [65]. Several miRNAs are differentially expressed in the failing heart [66, 67]. The empirical evidences from loss-, and gain-of function studies using different animal model systems suggest that miRNAs can alleviate progression of heart failure [68–71]. Recently, two miRNAs are used in clinical trials (anti-miR-122 for hepatitis C [72] and miR-34 mimic for primary liver cancer/tumor, clinicaltrials.gov # NCT01829971) corroborating their potential as a therapeutic target. DM is a microRNA-related disease [73, 74]. Through miRNA array analysis, we reported that majority of miRNAs are downregulated in Akita heart [75]. One of the downregulated miRNA was miR-133a, a cardioprotective and the most abundant miRNA in the heart [70, 76]. MiR-133a is also downregulated in streptozotocin-treated diabetic mice [10, 77, 78], indicating that genetic profiling from Akita heart may be useful for other models of T1DM. We found that miR-101c is upregulated (Fig 3C) and its target gene nebulin is downregulated in Akita heart (Fig 4C) supporting that downregulation of miRNA influences its target gene expression in Akita heart. LncRNA are another class of non-coding RNAs that plays regulatory roles in cardiovascular disease [79–81]. In the present study, we found differential expression of miRNAs (Fig 3C, complete list of miRNAs is uploaded on GEO website), and lncRNAs (Fig 4A and 4B) in Akita hearts. Although the unannotated lncRNA are open for future investigations for their potential roles in T1DM heart, it is reported that H19, which is upregulated in Akita hearts (Fig 4B), plays an important role in myoblast and myogenesis [82]. H19 is associated with regulation of a low fat-free mass [83], cardiac hypertrophy [84], and apoptosis [85]. The levels of H19 is downregulated in streptozotocin-treated Sprague-Dawley rat hearts [85]. The contrasting results in Akita mice and streptozotocin-treated Sprague-Dawley rats could be due to different species (rat vs mice) or T1DM model system (drug-induced vs spontaneous DM). Another lncRNAs that is downregulated in Akita hearts is Neat1 (Fig 4B). The role of Neat1 in the pathophysiology of the heart is unclear. However, reduced levels of Neat1 impairs myeloid differentiation in acute promyelocytic leukemia cells [86]. Future studies will reveal the specific roles of Neat1 in diabetic hearts.

In summary, our studies reveal several novel differentially expressed transcriptomes in Akita hearts and their implications in diabetic cardiomyopathy/heart failure. The novel candidates with unknown functions will encourage future studies to investigate their impact on diabetic cardiomyopathy/heart failure. Finding common differentially expressed transcriptomes by two methods (NGS and microarray) gives confidence that these genes are the prime candidates for investigating the underlying molecular mechanisms of diabetic heart failure. We also discovered several novel candidate lncRNAs, which set a platform to discover their potential roles in diabetic heart failure.
